# Supplementary material for: Describing the indescribable: A qualitative study of dissociative experiences in psychosis
Source: PLoS One. 2020 Feb 19;15(2):e0229091. doi: 10.1371/journal.pone.0229091 (PMC7029850; doi:10.1371/journal.pone.0229091)
Supplement: S1 File — (DOCX) [file pone.0229091.s001.docx]

# Supplementary material: Topic guide

**Topic Guide: Understanding Strange Feelings and Experiences**

Wherever <dissociation> is written, the interviewer will insert the participant’s language for describing dissociation. For example, if an individual talks about ‘spacing out’, the interviewer would ask “tell me about your experiences of spacing out”.

Please note that in accordance with good qualitative interview practice, the topic guide will be tailored to particular individuals. This will include mirroring the participant’s language, and encouraging story-telling via supportive prompts. As is common within interview studies, the interview process will be reviewed after an initial period.

The first few interviews (approx. 4) will be reviewed to discover early themes emerging from the data and used to adjust this topic guide to increase the accuracy and acceptability of the questions, as well as the breadth of data gained.

**Topic 1: Experiences of Dissociation**

*Thank you for coming today – I really appreciate you taking the time to meet with me. I am interested in your experiences of <dissociation>.*

*For the first part of the interview, could you tell me more about this? In the second part, I will have some questions to help me get some more detail about what you’ve told me. You don’t have to answer any questions that you don’t want to. What’s important to me is to really understand your experiences – so nothing is irrelevant. I will try and listen as much as possible and not interrupt you, but do ask me any questions you might have as we go along.*

*Please tell me about your experience(s) of <dissociation>.*

**Topic 2: Impact of the Dissociative Experiences**

*What was it like to experience <dissociation>?*

*What impact did it have on you?*

*Has this changed at all?*

**Topic 3: Relevant Factors**

*What do you think started the <dissociation>? / What brought on the <dissociation>?*

*What things kept it going?*

*Was there anything that helped bring it to a close?*

*Do you have any guesses about why it happened more than once?*

**Topic 4: Cognitive Appraisals***

*What do you remember thinking about <dissociation> while it was happening? / What goes through your mind when <dissociation> happens?*

*What goes through your mind when you first realise that <dissociation> is happening again?*

*What do you think about your <dissociation>?*

*Do you have any beliefs or opinions about <dissociation> that you can share with me?*

**Topic 5: Ending the Session**

*Is there anything I haven’t asked / we haven’t discussed about <dissociation> that you think is important for psychologists and researchers to know?*

*Is there something you’d like to talk about that we haven’t talked about?*

* Note that the interviewer should determine which thoughts / opinions / cognitive appraisals link with which specific symptoms of dissociation, since there is a large variation in dissociative experience, and these may elicit differing appraisals.
