# Supplementary material for: Describing the indescribable: A qualitative study of dissociative experiences in psychosis
Source: PLoS One. 2020 Feb 19;15(2):e0229091. doi: 10.1371/journal.pone.0229091 (PMC7029850; doi:10.1371/journal.pone.0229091)
Supplement: S2 File — (DOCX) [file pone.0229091.s002.docx]

# Supplementary material: Reflexive statement (EČ)

As described in the main manuscript text, I am a clinical psychologist carrying out a research programme into dissociation in psychosis, with experience working with (adult and first episode) psychosis and (child and adolescent) trauma in research and clinical settings. These experiences have given me insight into how distress may present across the lifespan, and specifically, how psychosis and trauma may present in the context of NHS services.

I work within a CBT-oriented psychology research group, and am supervised in this work by two researchers who are expert in CBT approaches for their specialty area of mental health. Therefore, I am aware that I favour CBT approaches in my work: indeed, two of the research questions in this study are explicitly framed in CBT terms. However, I am also familiar with a range of other psychological approaches and in particular often draw upon narrative and systemic perspectives when working therapeutically with clients.

My supervisors and I take a person-centred approach to research and clinical work. As a result, I strongly believe that listening closely to people’s lived experience is the most valid way to construct understanding for researchers and clinicians. It is this perspective that I have tried to hold at the forefront of my mind when analysing the data for this study.
